# Traditional Oriental Herbal Medicine for Children and Adolescents with ADHD: A Systematic Review

**DOI:** 10.1155/2012/520198

**Published:** 2012-12-17

**Authors:** Yuk Wo Wong, Deog-gon Kim, Jin-yong Lee

**Affiliations:** Department of Pediatrics, College of Korean Medicine, Kyung Hee University, 1 Hoegi-dong, Dongdaemun-gu, Seoul 130-702, Republic of Korea

## Abstract

*Objective*. To evaluate the efficacy of traditional Oriental herbal medicines (TOHM) for children and adolescents with ADHD. *Methods*. Randomized clinical trials published from January 1, 1990, to December 31, 2010, in English, Chinese, Japanese, or Korean language which evaluated the use of TOHM on ADHD subjects of 18 years old or below, diagnosed based on DSM-IV, were searched from MEDLINE, EMBASE, PsyINFO, Cochrane Library, and 10 other databases. *Results*. Twelve studies involving 1189 subjects met the inclusion criteria. In general, the included studies claimed that TOHM has similar efficacy to methylphenidate and at the same time has fewer side effects compared to methylphenidate. Some studies also suggested that the effect of TOHM sustained better than methylphenidate. However, solid conclusions could not be drawn because the included studies were not of high quality. Risk of bias issues such as randomization, allocation, concealment and blinding were not addressed in most of the studies, and the risk of publication bias could not be ruled out. *Conclusion*. Currently, there is not strong evidence to say that TOHM is effective in treating the core symptoms of ADHD.

## 1. Introduction

 Attention-deficit/hyperactivity disorder (ADHD) is a behavioral disorder of which patients display persistent pattern of inattention and hyperactivity/impulsivity or a combination of the two at an abnormal level that their social, academic, or occupational functioning is impaired [1]. While the etiology of ADHD is not clearly known, studies have suggested that the abnormality of the frontal network and dysregulation of catecholamines are the underlying pathophysiology [2]. Frontal lobes are involved in decision making to convert impulse to action, attention, and concentration, and they are primarily activated by the catecholamines, dopamine, and norepinephrine [3]. When frontal lobes are not fully activated, or when there are changes in the levels of dopamine and norepinephrine, the symptoms of hyperactivity and inattention are likely. 

When making a diagnosis of ADHD, clinicians should determine that the diagnostic criteria have been met by assessing information obtained from primarily parents, guardians, and teachers [4]. For adolescents, information from at least two teachers and other sources should be assessed because adolescents usually have multiple teachers and parents have little direct contact to observe their strengths and problems [4, 5]. Instruments such as the Revised Conners' Parent Rating Scale and Revised Conners' Teacher Rating Scale are useful for screening and assessing behavioral problems, and also helpful for assessing treatment effectiveness [6, 7].

 Behavioral therapy and pharmacotherapy are two kinds of treatments commonly used in ADHD. Recommendation of treatment for ADHD varies depending on the patient's age. While evidence-based behavioral therapy is recommended as the first line of treatment for preschool-aged children (4-5 years of age), school-aged children (6–11 years of age) and adolescents (12–18 years of age) are recommended a combination of medication and behavioral therapy [4].

 Stimulants are reported to be highly effective for most children in reducing the core symptoms of ADHD and thus are used as first line medication for ADHD patients [2, 4]. They are structurally similar to endogenous catecholamines and are thought to work by enhancing dopaminergic and noradrenergic neurotransmission [2, 8]. 

Methylphenidate (Ritalin) is the most commonly used stimulant for the treatment of ADHD [2, 9]. Around 70% of ADHD patients who receive stimulant treatment are given methylphenidate [10]. It is shown to be effective, at least in short term, on improving the core symptoms of ADHD such as attention, distractibility, and impulsivity (effect size 0.75–0.84, mean 0.78). Methylphenidate has observable effects on improving social and classroom behavior (effect size 0.63–0.85, mean 0.8) [9]. Pemoline (Cylert) is a stimulant that is longer acting than methylphenidate, but due to its potential for hepatotoxicity, it is regarded as a third line treatment [2]. In some cases, nonstimulants are also used in the treatment of ADHD such as the norepinephrine specific reuptake inhibitors atomoxetine, and antidepressants such as Imipramine, Phenelzine, and Bupropion [2, 11] but have been found to have significant differences in terms of efficacy compared to stimulants [8]. 

 While stimulants are effective for many children with ADHD, they may cause side effects, with the most common ones being decreased appetite, insomnia, and headache (Cohen's d 0.67, 0.40, and 0.33 resp.) [12]. Other side effects such as motor tics, abdominal pain, irritability, nausea, and fatigue are also reported [9]. Therefore, many parents of ADHD children try to search for more natural and safe treatment options [13, 14], which has resulted in a growing interest in complementary and alternative therapies (CAM), such as herbal remedies, dietary supplements, dietary modification, neurofeedback, homeopathic therapy, and chiropactic, in treatment of ADHD. Several surveys conducted on the use of CAM in ADHD showed over 50% of ADHD sufferers have used CAM [13, 15].

 Herbal medicine is a treatment measure used in traditional Oriental medicine. Although herb usually refers to materials from plant sources, in respect of traditional Oriental medicine, herbal materials can be originated from plants, animals, or minerals. In this review, traditional Oriental herbal medicine (TOHM) is defined as medicine made by materials used under traditional Oriental medical theory. Herbal materials that are not documented in the Korean Pharmacopoeia, the Japanese Pharmacopoeia, Pharmacopoeia of the People's Republic of China, Zhong Hua Ben Cao, and Zhong Yao Da Ci Dian (Chinese Medical Great Dictionary) are considered outside the context of TOHM. TOHM should be taken orally and studies employing other route of administration, such as intravenous or transdermal, are excluded from this review. 

 Even though ADHD was not described in literature of traditional Oriental medicine, in traditional Oriental medical theory, ADHD is related to congenital deficiency or insufficient postnatal nourishment that leads to imbalances in the body. It is suggested that the disorder is related to the heart, the liver, the spleen, and the kidneys [16]. TOHM is believed to work by adjusting the inner imbalances of ADHD patients and thereby relieving the symptoms.

 A study on ADHD using an animal model of spontaneous hypertensive rat treated with an Oriental herbal decoction comprised of Caulis Polygoni Multiflori (stem of *Polygonum multiflorum* Thunb.), Radix Rehmanniae Preparata (processed Rehmannia root), Carapax et Plastrum Testudinis (Carapace and plastron of *Chinemys reevsii* (Gray)), Os Draconis (fossilized bones), Radix Polygalae (Polygala root), and Rhizoma Acori Tatarinowii (Grassleaf Sweetflag Rhizome) showed that the decoction increased the amount of dopamine at the frontal cortex and corpus striatum [17], suggesting that its possible mechanism in ADHD is to increase dopamine level and thereby enhance catecholaminergic neurotransmission.

This review aims to evaluate the efficacy of TOHM as a treatment for ADHD in patients under the age of 18. TOHM is natural and often perceived to have fewer side effects than conventional ADHD pharmacotherapy. Various research and clinical studies have been conducted on TOHM's efficacy on ADHD, but very few articles review the evidence of efficacy of the treatment. A systematic review on complementary medicines for ADHD suggested that a Chinese herbal medicine may be effective for ADHD [18] however in the review only one study about Chinese herbal medicine was included and analyzed. Further compilation and analysis of currently available data about TOHM on ADHD may help to understand the true effect of the treatment on the disorders, and provide insight into the direction of future research. 

## 2. Methods

### 2.1. Database Searching

 English, Chinese, Korean and Japanese articles on randomized clinical trials (RCTs) of Oriental herbal treatment on ADHD published between January 1, 1990, and December 31, 2010, were searched from various databases. The details of search terms used in different databases are presented in the appendix. The following databases were searched:

1. Cochrane Library,
2. EMBASE,
3. MEDLINE,
4. AMED,
5. CINAHL Plus,
6. PsyINFO,
7. SinoMed–CBM—Chinese Database,
8. China Journal Net—Chinese Database,
9. WanFang Data—Chinese Database,
10. Oriental Medicine Advanced Searching Integrated System (OASIS)—Korean Database,
11. Scholarly and Academic Information Navigator (CiNii)—Japanese Database,
12. Database of Grants-in-Aid for Scientific Research (KAKEN)—Japanese Database,
13. Japanese Institutional Repositories Online (JAIRO)—Japanese Database,
14. Academic Research Database Repository (NII-DBR)—Japanese Database.

### 2.2. Reference List

 Other than searching from databases, the reference lists of the included studies were referred to in order to identify more potential articles.

### 2.3. Criteria for Considering Studies for This Review

#### 2.3.1. Type of Studies

Randomized clinical trials of TOHM. The efficacy of TOHM treatment should be compared to either a placebo or a conventional medication used for treating ADHD. If there was a baseline treatment, it had to be the same in both the treatment and control groups. Studies only comparing different TOHM formulae, or comparing TOHM with other traditional Oriental treatment such as acupuncture were excluded. Studies without indicating “randomized" were considered not randomized and excluded.

#### 2.3.2. Type of Participants

Subjects under the age of 18 who were diagnosed with ADHD based on DSM-IV.

#### 2.3.3. Type of Interventions

 Traditional Oriental herbal medicine must be used. Herbs that are not documented in the Korean Pharmacopoeia, the Japanese Pharmacopoeia, Pharmacopoeia of the People's Republic of China, Zhonghua Bencao, and Zhongyao Dacidian were not considered. Other treatment measures of Oriental medicines such as acupuncture and moxibustion were excluded.

#### 2.3.4. Types of Outcome Measures

 The core symptoms of ADHD (hyperactivity, impulsivity, and inattention) were considered in this review. Core symptoms should be assessed by at least one of the following tools: Revised Conners' Parent Rating Scale, Revised Conners' Teacher Rating Scale, Conners' Hyperactivity Index, Conners' Abbreviated Symptoms Questionnaire, Conners' Global Index for Parents, and/or Conners' Global Index for Teachers. 

### 2.4. Risk of Bias Assessment of Included Studies

 The risk of bias of all the included studies was assessed according to Cochrane Handbook for Systematic Reviews of Invention version 5.1.0.

## 3. Results

**Figure 1:**
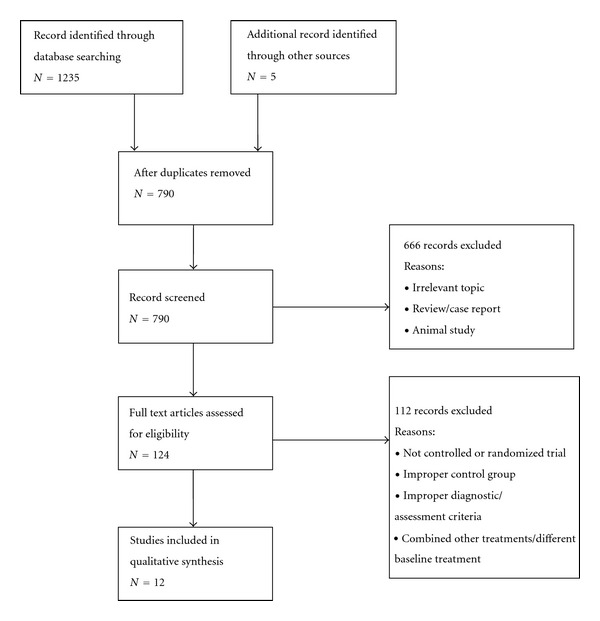
Selection of studies flowchart.

The search came up with 1240 results, and 12 studies [16, 19–29] involving 1189 subjects were included in this review (see Figure 1 for included studies selection).

 All of the studies included in this review were conducted in China as single-centre trials. Five results in Japanese and eighteen results in Korean were identified. Only one Japanese article was about a clinical trial; however the trial was not a randomized trial and was therefore not selected.

 Among the twelve included studies, none included the information on how sample size was derived and whether the study was statistically powered. The length of study ranged from 4 weeks to 24 weeks. Six studies had follow-up observation on subjects, ranging from 2 weeks to 12 months after finishing treatment, to evaluate whether the intervention sustained effectiveness after treatment is stopped while the other six studies did not report if follow-up observations were conducted. Ten of the included studies reported homogeneity of baseline characteristics, but only seven [20, 22, 24, 26–29] showed relevant descriptive statistical data. Two studies [16, 19] did not report if baseline characteristics of subjects were homogenous. Only one of the studies [27] specified the subtype of ADHD subjects included in the study. Characteristics of included studies are summarized in Table 1.

### 3.1. Assessment of Risk of Bias

 In general, the risk of bias in the included articles is unclear. Very limited information was revealed in the studies to enable the reviewers to tell if the included studies were at risk of bias.

 Only one of the included studies [16] described how randomization was done, but the study used two randomization methods where part of the subjects were randomized using a random number table while part were allocated to the treatment or control groups by their patient record numbers. The allocation concealment issue was not addressed in any of the included studies. 

 The blinding method was also not addressed in most of the studies, and only two of the included studies [22, 26] claimed to be a double-blind trial. Li et al. (1999) described the blinding method, which was to include a placebo resembling methylphenidate in the treatment group and a placebo that looked like the corresponding TOHM in the control group. Wang et al. (2003) did not describe how blinding was done. 

 Most of the studies indicated no missing data. However, Ma et al. (2007) [25] did not specify the initial number of subjects so it could not be determined if there was any participant drop-out. Ma et al. (2007) [16] reported seven drop-outs but no explanations were provided, and it was unclear whether the drop-outs were from the treatment group or the control group. In another study [23], three subjects were excluded and there were five drop-outs, but the reasons were not sufficiently provided. Among those eight subjects, it is only known that four of the drop-outs were reported to have terminated the study due to adverse effect of methylphenidate. Those three studies were considered to have unknown risk of bias on incomplete outcome data.

 Study protocols were not available for any of the included studies, therefore it could not be discerned whether all pre-specified outcomes were reported. Lai and Li (2006) [21] did not report the baseline score before treatment and score after treatment. Xu (2005) [27] did not report the baseline score of rating. The two studies were considered to have high risk of reporting bias.

**Figure 2:**
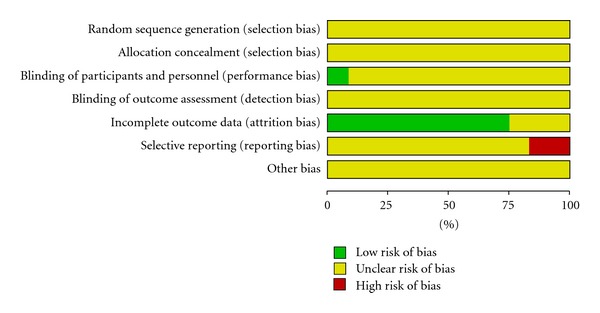
Risk of bias graph of the included studies.

**Figure 3:**
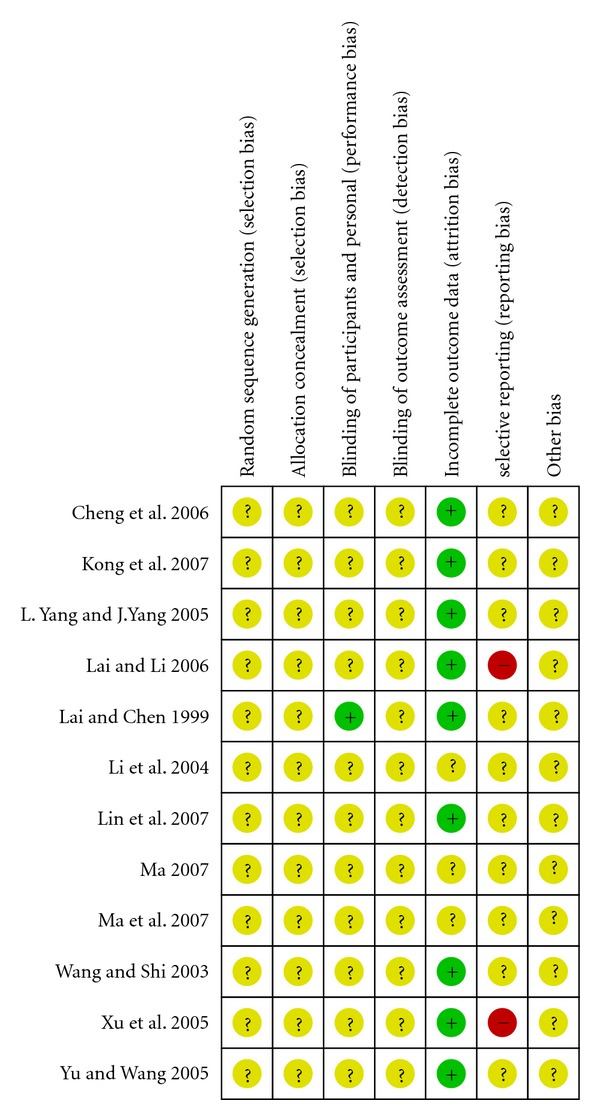
Risk of bias summary of the included studies. −: high risk of bias, +: low risk of bias, ?: unknown risk of bias.

 The risk of bias graph and summary are presented in Figures 2 and 3, respectively.

### 3.2. Diagnosis and Assessment of the Disorder

 Although all included studies specified the diagnostic criteria and method to assess treatment effect, only one study [16] specified who completed the rating questionnaire or did the rating assessment. None of the studies addressed under which setting the assessment was done. Also, the language of assessment tools was not specified. It was not clear if the assessment tools were validated in cases where they were translated into another language.

### 3.3. Treatment Effectiveness

 The herbal formulae used in the included twelve studies varied, and dosage forms of decoction, granules, oral liquids, pill, and so forth were used. Nine studies [16, 19–22, 25, 27–29] provided the ingredients of the formulae, but among them five [22, 25, 27–29] did not specify the amount of each herb used in the formula, and therefore there was very little information on drug to extract ratio. Three studies [23, 24, 26] did not provide the formula at all. None of the studies mentioned how the herbal medicines used were standardized. The details of the herbal ingredients used in the included studies, their dosage form, and daily dose are presented in Table 2.

 Among the twelve studies, there were ten 2-arm studies [16, 19–23, 26–29] that compared the effectiveness of TOHM to methylphenidate, and one 3-arm study [24] which included a TOHM treatment group, a TOHM control group, and methylphenidate control group. These studies claimed that TOHM had no significant difference on effectiveness compared to methylphenidate. However, seven of them [16, 21, 22, 26–29] did not conduct statistical analyses to demonstrate whether there was significant efficacy compared to the baseline.

 The remaining one of the twelve included studies [24] evaluated the net effect of TOHM by having a combined treatment group of TOHM and methylphenidate, and a methylphenidate control group. The study reported that TOHM and methylphenidate worked better in combination than methylphenidate alone, with statistically significant differences when comparing the rate of effectiveness of the two groups.

### 3.4. Follow-Up Observation on Effectiveness

 Among the included studies, six had follow-up observations to evaluate the sustainability of treatment effect of core symptom after stopping medication, while the other six studies did not specify if follow-up observation was done. The follow-up period varied from 2 weeks to 12 months after stopping the treatment. For the six studies with follow-up observation, five [20, 23, 24, 27, 28] compared the treatment effect of TOHM and methylphenidate after stopping medication and all reported the effect of TOHM sustained better compared to methylphenidate. The remaining one [25] only followed up on the effect of the TOHM treatment group.

### 3.5. Safety and Side Effects

 In general, TOHM was claimed to have fewer side effects than methylphenidate. Eight of the included studies [19–24, 27, 28] discussed side effects. In general, more cases of side effect were reported in the methylphenidate control group than the TOHM treatment group. Two studies [26, 28] reported no cases of side effect in the TOHM treatment group. Cheng et al. (2006) reported that among the side effect cases in their study, dry mouth, sweating, nausea, weight loss, loss of appetite, and headache were significantly fewer in the TOHM treatment group compared to the methylphenidate control group (*P* < 0.05) [19]. Lai and Li (2006) reported that cases of loss of appetite and drowsiness were significantly fewer (*P* < 0.05) [21]. Lin et al. (2007) reported that cases of sleeplessness, dizziness/headache, sweating, dry mouth, nausea, loss of appetite, weight loss, and constipation were significantly fewer in the treatment group than the control group (*P* < 0.01) [24]. Xu (2005) compared the average score of Treatment Emergent Symptoms Scale (TESS) in the treatment group and the control group, and reported that the average score was significantly higher in the control group (*P* < 0.01) [27]. Four other studies [19, 20, 22, 26] also claimed to have used TESS to evaluate side effects but scores were not reported.

 Three studies [16, 25, 28] performed liver function tests, renal function tests, and/or ECGs on subjects after treatment and results showed that subjects had no impairment on liver function, renal function, and/or cardiac function after treatment with TOHM and methylphenidate, suggesting that both TOHM and methylphenidate do not cause any significant safety concern on treatment of ADHD, at least in the short term.

## 4. Discussion

 This review included twelve studies, and though findings of this review suggested that the herbal preparations covered under the term TOHM may be effective in treating the core symptoms of ADHD; the overall evidence is not strong enough to draw solid conclusions, because in general the clinical trials were not of high quality and the herbal preparations far too different. Additionally, it cannot be ruled out that there is possibility of publication bias.

 The included studies that discussed side effect issues all suggested that TOHM had fewer side effects compared to methylphenidate. However, such result should be interpreted with caution, because first of all it was not clear whether the side effect cases, both in the TOHM group and the methylphenidate group, were investigated to find out if they were related to the intervention. Secondly, it was not addressed in most studies whether blinding was done. As mentioned, since TOHM is indigenous to the study population, and is often perceived as natural with fewer side effects, if measures are not properly done to blind subjects from knowing what treatment they are getting, it may cause bias in reporting side effects.

 In the future, should more clinical trials on ADHD using TOHM as treatment be conducted, clinical investigators should consider to address the issues discussed below in order to improve the robustness of data.

 In the diagnosis of ADHD and assessment of treatment efficacy, tools such as rating scales are often used. In order to make precise assessment, information should be obtained from different parties including parents, guardians, and teachers under different settings, such as home and school [4]. However, among the included studies, eleven of them did not specify who completed the questionnaires for assessment or under which setting the assessment was done. It was difficult to tell whether sufficient information was obtained to facilitate an accurate assessment of the treatment effect. Also, since the studies were done in Chinese population, it was possible the questionnaires used were in Chinese, but no information was available to tell whether the questionnaires, in case written in another language, were validated or not. Investigators did not indicate whether the tool of assessment has been modified to suit the study purpose as well. Such information should be described in the study methods.

 Among the included studies, the herbal medicines themselves varied. Some of the studies did not tell what herbs were used, some did not specify the amount of herbs used, and the treatment dosage was not clear in some studies. Also, the treatment period varied for each study. Due to such heterogeneity, it is not possible to deduce from the data which herbal formulae or ingredients may be effective for ADHD, nor to conclude that TOHM is effective for ADHD in children and adolescents. Although it is inevitable that different studies may use different herbs and have different treatment periods, the materials used and the amount should be stated clearly in the publication of clinical trial results. As various herbal treatments are used in different clinical studies the results even of the positive studies cannot be compared. Clinical investigators may have to consider repeating a study with the same herbal treatment, or to conduct a clinical trial in multiple sites.

 Due to the complex nature of herbs, how the consistency of herbal treatment is maintained throughout a clinical study is often an issue to consider. Most of the studies included in this review used the herbal treatment substances in form of decoctions or other preparations such as granules or oral liquid prepared by the clinical sites. It was not addressed how the consistency of treatment substances was kept throughout the studies, or how the treatment substances were standardized to ensure quality. In addition, in three of the studies [16, 21, 29], prescriptions given to subjects varied according to their symptoms. Although one of the characteristics of traditional Oriental medicine is tailor-made treatment according to the patient's condition, in a clinical study setting, this may introduce confounding variables. Investigators should make an effort to ensure the herbal treatment used in a study is of consistent quality throughout the study period. One of the possible ways to address this problem is to use herbal medicines prepared by qualified pharmaceutical manufacturers, and the treatment preparation should also be standardized.

### 4.1. Strength of This Review

In this review, the reviewers performed a thorough search in various databases. Other than major databases that have information of articles published mostly in English, additional Chinese, Korean, and Japanese databases were searched to identify potential studies. Articles written in English, Chinese, Korean, and Japanese languages were screened in order to include as many suitable studies in the review as possible.

### 4.2. Limitation of This Review

Due to limited resources, the reviewers could only seek published studies. For a robust review, nonpublished data should also be sought. Also, the reviewers were not able to contact the authors of included studies for clarification and further information on their studies.

 Even though the reviewers did a thorough search of published studies, the included studies were all conducted on Chinese population. Little could be told about the effect of TOHM on ADHD on populations of other countries.

## 5. Conclusion

 This review included twelve studies on different herbal preparations from TOHM as a treatment for children and adolescents with ADHD. Findings suggest that some of them may have similar efficacy to methylphenidate, but solid conclusions could not be drawn due to quality problems of the clinical trials. In conclusion, currently there is no strong evidence to suggest that TOHM is effective in treating the core symptoms of ADHD. More studies with low risk of bias and using the same herbal preparation are required before further conclusions can be drawn. 

## Figures and Tables

**Table 1:** Selected studies characteristics.

Study	Method	Participants	Intervention	Outcomes
Cheng et al. 2006 [19]	Randomized Control Trial	*Treatment group* Number: 50, male/female: 39/11 Age range: mean 8.7 ± 1.5 *Control group* Number: 50, male/female: 40/10 Age range: mean 8.9 ± 1.4 Country: China Diagnostic criteria: DSM-IV Baseline characteristic: not described	*Treatment group* Yizhiyidong decoction 30 mL each time, 3 times a day for 12 weeks *Control group* Ritalin 5 mg–10 mg each time, twice a day for 12 weeks	The study defined treatment effect as follows: (i) Showing effectiveness—disappearance of symptoms, Conners index decrease ≥80%: treatment/control: 16/23 (ii) Showing improvement—improvement of symptoms, Conners index decrease 80%–50%: treatment/control: 21/18 (iii) Ineffective—no improvement or worsening in core symptoms, Conners index and studying: treatment/control: 13/9

Kong et al. 2007 [20]	Randomized Control Trial	*Treatment group* Number: 60, male/female: 44/16 Age range: mean 8.4 ± 1.42 *Control group* Number: 60, male/female: 48/12 Age range: mean 8.40 ± 1.40 Country: China Diagnostic criteria: DSM-IV Baseline characteristic: homogeneity for gender, age, and IQ	*Treatment group* Qijudihuang pill, 3 pills, 3 times a day for 3 months *Control group* Ritalin starting from 5mg and adjusted to 10 mg–20 mg for 3 months	There was no significant difference of treatment effectiveness between treatment group and control group. Followup 6 months and 12 months after treatment indicated that treatment had significantly more sustainable effect than control, and statistics showed that treatment group has significantly less side effects compared to control group.

Lai and Li 2006 [21]	Randomized Control Trial	Treatment/control: 21/19 Male/female: 24/16 Age range: 6–14 (mean 8.437 ± 2.061) Country: China Diagnostic criteria: DSM-IV Baseline characteristic: homogeneity for gender, age, and course of disease	*Treatment group* Oriental herbal medicine decoction 150 mL a day, 5 days a week for 8 weeks *Control group* Ritalin 0.3–0.5 mg/kg a day, 5 days a week for 8 weeks	The study defined treatment effect as follows: (i) Recovery—disappearance of core symptoms, Conners index decrease >80%, obvious improvement in studying, effectiveness sustains after stopping medication for 6 months: treatment/control: 2/1 (ii) Showing effectiveness—obvious alleviation of core symptoms, Conners index decrease >50%, improvement in studying: treatment/control: 10/9 (iii) Showing improvement—improvement of core symptoms, Conners index decrease >30%, improvement in studying but not stable: treatment/control: 7/6 (iv) Ineffective—no improvement or worsening in core symptoms, Conners index and studying: treatment/control: 3/3

Li and Chen 1999 [22]	Randomized Control Trial	*Treatment group* Number: 37, male/female: 37/0 Age range: mean 10.7 ± 4.2 *Control group* Number: 33, male/female: 33/0 Age range: mean 10.3 ± 3.9 Country: China Diagnostic criteria: DSM-IV and Conners Rating Scale Baseline characteristic: Homogeneity for gender, age, course of disease, and IQ	*Treatment group* Duodongning granule 3 g for age <8, 6 g for age ≥8 daily with placebo that resembles Ritalin, 1 tablet each day, 6 days a week for 4 weeks *Control group* Ritalin 10 mg daily, with placebo that resembles Duodongning granule and same dosage as treatment group, 6 days a week for 4 weeks	The study defined treatment effect as follows: (i) Showing effectiveness—disappearance of core symptoms, Conners index <1.2, obvious improvement in studying, normal social function: treatment/control: 17/15 (ii) Showing improvement—improvement of core symptoms, Conners index decrease but still >1.5, improvement in studying and social function but not stable: treatment/control: 16/14 (iii) Ineffective—no evident improvement in core symptoms, Conners index, studying, and social function: treatment/control: 4/4 3 subjects in treatment group reported mild loss of appetite, while 7 subjects in control group reported evident loss of appetite and 1 subject reported sleeplessness.

Li et al. 2004 [23]	Randomized Control Trial	Treatment/control: 58/48 Male/female: 84/22 Age range: 7–15 Country: China Diagnostic criteria: DSM-IV Baseline characteristic: Homogeneity for age, gender, and course of disease There were 114 subjects originally, 3 excluded and 5 attrited. 4 were attrited due to adverse effect caused by methylphenidate but attrition/exclusion reason for other subjects was not indicated.	*Treatment group* Yizhiningshen granules 1 dose for age 7–10, 2 doses for age 10–15, twice a day for 6 weeks *Control group* Ritalin 5 mg per day for age 7–8, 10 mg per days for age 8–15, twice a day, 5 days a week for 6 weeks	The study defined treatment effect as follows: (i) Recovery—disappearance of core symptoms, Conners index decrease >80%, obvious improvement in studying, effectiveness sustains after stopping medication for 6 months, Chinese medicine therapeutic index ≥90%: treatment/control: 6/2 (ii) Showing effectiveness—obvious alleviation of core symptoms, Conners index decrease ≤80% and >50%, improvement in studying, Chinese medicine therapeutic index <90% and ≥60%: treatment/control: 20/18 (iii) Showing improvement—improvement of core symptoms, Conners index decrease ≤50% and >30%, improvement in studying but not stable, Chinese medicine therapeutic index <60% and ≥10%: treatment/control: 26/22 (iv) Ineffective—no improvement or worsening in core symptoms and studying, Conners index decrease ≤30%, Chinese medicine therapeutic index <10%: treatment/control: 6/6

Lin et al.2007 [24]	Randomized Control Trial	*Treatment group* Number: 40, male/female: 32/8 Age range: 6.5–13, mean 8.7 ± 2.7 *Control group 1 *(*Ritalin*) Number: 40, male/female: 31/9 Age range: 7–12, mean 8.5 ± 2.5 *Control group 2* (*treatment* + *Ritalin*) Number: 40, male/female: 32/8 Age range: 7–11, mean 8.2 ± 2.1 Country: China Diagnostic criteria: DSM-IV Baseline characteristic: Homogeneity for gender, age, and course of disease	*Treatment group* Ningshen oral liquid 30–60 mL per day for 12 weeks *Control group 1* Ritalin 5 mg–40 mg per day for 12 weeks *Control group 2* Ningshen oral liquid 30–60 mL plus Ritalin 5 mg–40 mg per day for 12 weeks	The study defined treatment effect as follows: (i) Near recovery—disappearance of core symptoms, Conners index decrease >80%, obvious improvement in studying: treatment/control 1/control 2: 4/3/7 (ii) Showing effectiveness—obvious alleviation of core symptoms, Conners index decrease ≤80% and >50%, improvement in studying: treatment/control 1/control 2: 7/7/10 (iii) Showing improvement—improvement of core symptoms, Conners index decrease ≤50% and >30%, improvement in studying but not stable: treatment/control 1/control 2: 16/19/17 (iv) Ineffective—no improvement or worsening in core symptoms and studying, Conners index decrease ≤30%: treatment/control 1/control 2: 13/11/6 Assessment of Conners index 12 weeks after stopping medication reported that there was significant drop in effectiveness in Ritalin control group but not in treatment group and combined treatment group.

Ma 2007 [16]	Randomized Control Trial	*Treatment group* Number: 22, male/female: 15/7 *Control group* Number: 20, male/female: 20/16 Age range: 6–13 Country: China Diagnostic criteria: DSM-IV Baseline characteristic: not described There were originally 49 subjects in the study and 7 cases were attrited, but there was no indication of whether they belonged to treatment group and control group. Reasons for attrition were also not sufficiently presented	*Treatment group* Duodongting decoction 150 mL each time for age 4–7, 200 mL each time for age 8–12, twice a day for 28 days, and break for 2 days (1 treatment cycle) before starting another treatment cycle, for 2 cycles + behavioral therapy *Control group* Ritalin 0.45 mg/kg for 28 days and break for 2 days (1 treatment cycle) before starting another treatment cycle, for 2 cycles + behavioral therapy	The study defined treatment effect as follows: (i) Showing effectiveness—hyperactivity, and inattention alleviated by 2/3: treatment/control: 7/6 (ii) Showing improvement—obvious symptomatic improvement, hyperactivity and inattention alleviated by 1/2: treatment/control: 11/10 (iii) Ineffective—no symptomatic relief after 2 treatment cycles treatment/control: 4/4

Ma et al. 2007 [25]	Randomized Control Trial	Treatment group/control group-Chinese medicine/control group-Ritalin: 55/53/51 Country: China Diagnostic criteria: DSM-IV Baseline characteristic: homogeneity for age, gender, and course of disease Attrition criteria was mentioned but there was no indication if any subject was attrited because initial number of subjects randomized was not stated	*Treatment group* YIzhiningshen granules 10 g each time for age 6–10, 15 g each time for age 11–18, twice a day for 24 weeks *Control group 1-Chinese medicine * Jingning oral liquid 10 mL each time, twice a day, 5 days per week for 24 weeks *Control group 2-Ritalin* Ritalin 5 mg each time for age 6–8, and 10 mg each time for age 9–18, twice a day, 5 days per week for 24 weeks	The study defined treatment effect as follows: (i) Recovery—disappearance of core symptoms, Conners index decrease >80%, obvious improvement in studying and social function, Chinese medicine therapeutic index ≥90%: treatment/control 1/control 2: 6/3/3 (ii) Showing effectiveness—obvious alleviation of core symptoms, Conners index decrease ≤80% and >50%, improvement in studying and social function, Chinese medicine therapeutic index <90% and ≥60%: treatment/control 1/control 2: 29/18/19 (iii) Showing improvement—improvement of core symptoms, Conners index decrease ≤50% and >30%, improvement in studying but not stable, Chinese medicine therapeutic index <60% and ≥10%: treatment/control 1/control 2: 15/27/22 (iv) Ineffective—no improvement or worsening in core symptoms and studying, Conners index decrease ≤30%, Chinese medicine therapeutic index <10%: treatment/control 1/control 2: 5/5/7

Wang and Shi 2003 [26]	Randomized Control Trial	*Treatment group* Number: 58, male/female: 49/9 Age range: mean 9.3 ± 2.9 *Control group* Number: 50, male/female: 42/8 Age range: mean 9.5 ± 3.2 Country: China Diagnostic criteria: DSM-IV and Conners index >1.5 Baseline characteristic: homogeneity for age, gender, course of disease, and IQ	*Treatment group* Jingning oral liquid 10 mL each time, twice a day for 4 weeks *Control group* Ritalin 10 mg in the morning and 5 mg at night for 4 weeks	The study defined treatment effect as follows: (i) Showing effectiveness—disappearance of symptoms, obvious improvement in social function and studying, Conners index < 1.2: treatment/control: 27/23 (ii) Showing improvement—improvement of symptoms, improvement in social function and studying but improvement not stable, decrease in Conners index but still >1.5: treatment/control: 26/22 (iii) Ineffective—no improvement or worsening in symptoms, Conners index and studying: treatment/control: 5/5 No side effects observed in treatment group but 10 subjects in control group reported nausea and loss in appetite, 2 subjects reported dizziness, and 1 subject reported sleeplessness.

Xu 2005 [27]	Randomized Control Trial	*Treatment group* Number: 100, male/female: 87/13 Age range: mean 9.3 ± 2.4 *Control group* Number: 50, male/female: 43/7 Age range: mean 9.1 ± 2.3 Country: China Diagnostic criteria: DSM-IV criteria of combined type ADHD Baseline characteristic: homogeneity for age, gender, course of disease, and IQ	*Treatment group* Jingningzhidong granules 1 g/kg/day, maximum 50 g, for 12 weeks *Control group* Ritalin 0.3 mg/kg/day, increase dosage gradually to maximum 0.6 mg/kg/day, for 12 weeks	The study defined treatment effect as follows: (i) Showing effectiveness—decrease in Conners index by ≥66%: treatment/control: 56/26 (ii) Showing improvement—decrease in Conners index ≥33% and <66%: treatment/control: 22/14 (iii) Ineffective—decrease in Conners index < 33%: treatment/control: 22/10 There was almost no side effect reported in treatment group but subjects in control group reported more obvious and persistent side effects. Assessment of Conners index 4 weeks after stopping medication reported that there was significant drop in effectiveness in control group but not in treatment group.

L. Yang and J. Yang 2005 [28]	Randomized Control Trial	*Treatment group* Number: 48, male/female: 40/8 Age range: 6–12 (mean 8.3) *Control group* Number: 38, male/female: 31/7 Age range: 6.5–13 (mean 8.6) Country: China Diagnostic criteria: DSM-IV Baseline characteristic: Homogeneity for gender, age, course of disease, and coexisting symptoms	*Treatment group* Yizhiningshen oral liquid 2 × 10 mL, 3 times a day for 3 months + behavioral therapy *Control group* Ritalin 10 mg, once per day for 3 months, no medication during weekend and holiday + behavioral therapy	The study defined treatment effect as follows: (i) Recovery—disappearance of core symptoms, Conners index decrease >80%, obvious improvement in studying, effectiveness sustains after stopping medication for 6 months: treatment/control: 6/2 (ii) Showing effectiveness—obvious alleviation of core symptoms, Conners index decrease >50%, improvement in studying: treatment/control: 16/15 (iii) Showing improvement—improvement of core symptoms, Conners index decrease >30%, improvement in studying but not stable: treatment/control: 20/17 (iv) Ineffective—no improvement or worsening in core symptoms, Conners index and studying: treatment/control: 6/4 Treatment group showed improvements in co-existing symptoms such as loss in appetite, recurrent flu, but no evident improvement in control group.

Yu and Wang 2005 [29]	Randomized Control Trial	*Treatment group* Number: 68, male/female: 54/14 Age range: mean 9.1 ± 2.8 *Control group* Number: 20, male/female: 15/5 Age range: mean 9.3 ± 2.7 Country: China Diagnostic criteria: DSM-IV and Conners index > 1.5 Baseline characteristic: homogeneity for age, gender, and course of disease	*Treatment group* Jingning decoction one dose per day for 3 months *Control group* Ritalin 0.2–0.5 mg/kg/day for 3 months	The study defined treatment effect as follows: (i) Showing effectiveness—disappearance of symptoms, obvious improvement in social function and studying, Conners index < 1.2: treatment/control: 42/15 (ii) Showing improvement—improvement of symptoms, improvement in social function and studying but improvement not stable, decrease in Conners index but still >1.5: treatment/control: 19/3 (iii) Ineffective—no improvement in symptoms, Conners index and studying: treatment/control: 7/2

**Table 2:** Details of the herbal treatments used in the included studies.

Cheng et al. 2006 [19]	*Ingredients and amount*: Radix Rehmanniae Preparata 6 g, Carapax et Plastrum Testudinis 5 g, Cervi Cornu Degelatinatum 5 g, Rhizoma Acori Tatarinowii 10 g, Radix Polygalae 10 g, Radix et Rhizoma Salviae Miltiorrhizae 10 g, Fructus Schisandrae Chinensis 5 g *Dosage form*: Decoction. The above amount of ingredients are boiled with water to yield 90 mL of decoction. *Daily dose*: 90 mL *Standardization*: not known

Kong et al. 2007 [20]	*Ingredients and amount*: Fructus Lycii 9 g, Flos Chrysanthemi 9 g, Radix Rehmanniae Preparata 24 g, Fructus Corni 12 g, Rhizoma Dioscoreae 12 g, Rhizoma Alismatis 9 g, Cortex Moutan 9 g, Poria 9 g *Dosage form*: Pills. It was not clear how many pills were made out of the above amount of herbs. *Daily dose*: 9 pills *Drug to extract ratio*: not known *Standardization*: not known

Lai and Li 2006 [21]	*Ingredients and amount*: Os Draconis 20 g, Carapax et Plastrum Testudinis 10 g, Radix Polygalae 5 g, Rhizoma Acori Tatarinowii 10 g, Fructus Tritici Levis 20 g, Radix Ophiopogonis 10 g, Caulis Polygoni Multiflori 15 g, Radix *Codonopsis* 15 g, Poria 15 g, Radix Rehmanniae Preparata 15 g, Fructus Schisandrae Chinensis 4 g, Radix et Rhizoma Glycyrrhizae 4 g. Depending on the symptoms of different patients, other herbs might be added but the amount was not specified. *Dosage form*: Decoction. 150 mL of decoction was made from the above ingredients. *Daily dose*: 150 mL *Standardization*: not known

Li and Chen 1999 [22]	*Ingredients and amount*: Fructus Lycii, Radix Rehmanniae Preparata, Fructus Schisandrae Chinensis, Radix et Rhizoma Ginseng, Poria, Radix et Rhizoma Glycyrrhizae. Amount was not specified. *Dosage form*: Granules *Daily dose*: 3 g or 6 g depending on the age of subject *Drug to extract ratio*: not known *Standardization*: not known

Li et al. 2004 [23]	*Ingredients and amount*: not known *Dosage form*: Granules *Daily dose*: 2 doses or 4 doses depending on the age of subject. Amount of granules contained in 1 dose was not specified. *Drug to extract ratio*: not known *Standardization*: not known

Lin et al. 2007 [24]	*Ingredients and amount*: not known *Dosage form*: oral liquid *Daily dose*: 30–60 mL *Drug to extract ratio*: not known *Standardization*: not known

Ma 2007 [16]	*Ingredients and amount*: Flos Magnoliae 10 g, Radix Paeoniae Alba (parched) 30 g, Rhizoma Gastrodiae 8 g, Radix Isatidis 15 g, Radix Scrophulariae 15 g, Massa Medicata Fermentata 6 g, Fructus Crataegi 6 g *Dosage form*: Decoction. The amount of decoction yielded from the above ingredients was not specified. *Daily dose*: 150 mL or 200 mL depending on the age of subject *Drug to extract ratio*: not known *Standardization*: not known

Ma et al. 2007 [25]	*Ingredients and amount*: Homonis Placenta, Radix Rehmanniae Preparata, Rhizoma Acori Tatarinowii, Radix Polygalae, Rhizoma Alismatis, Rhizoma Coptidis, and so forth. Amount was not specified. *Dosage form*: Granules *Daily dose*: 20 g or 30 g depending on the age of subject *Drug to extract ratio*: not known *Standardization*: not known

Wang and Shi 2003 [26]	*Ingredients and amount*: not known *Dosage form*: Oral liquid *Daily dose*: 20 mL *Drug to extract ratio*: not known *Standardization*: not known

Xu 2005 [27]	*Ingredients and amount*: Radix Bupleuri, Radix Peoniae Alba, Ramulus cum Uncis Uncariae, Os Draconis, Margaritifera Concha, Radix Pseudostellariae, Fructus Alpiniae Oxyphyllae, Radix Polygalae, Rhizoma Acori Tatarinowii, Fructus Schisandrae Chinensis, Poria, Radix et Rhizoma Glycyrrhizae Preparata. Amount was not specified. *Dosage form*: Granules. 1 g of granules is equivalent to 5 g of herbs. *Daily dose*: up to 50 g depending on the body weight of subject *Standardization*: not known
L. Yang and J. Yang 2005 [28]	*Ingredients and amount*: Radix Rehmanniae Preparata, Radix Astragali, Radix Peoniae Alba, Os Draconis, Radix Polygalae, Rhizoma Acori Tatarinowii, Fructus Schisandrae Chinensis, Rhizoma Atractylodis Macrocephalae. Amount not specified. *Dosage form*: Oral liquid *Daily dose*: 60 mL *Standardization*: not known

Yu and Wang 2005 [29]	*Ingredients and amount*: Rhizoma Coptidis, Pericarpium Citri Reticulatae, Rhizoma Pinelliae Preparatum, Poria, Rhizoma Atractylodis Macrocephalae, Radix Peoniae Alba, Ramulus cum Uncis Uncariae, Flos Chrysanthemi, Radix Polygalae, Fructus Alpiniae Oxyphyllae, Fructus Corni. Amount was not specified. Other herbs might be added depending on the symptoms of individual subject but the exact ingredients and amount were not given. *Dosage form*: Decoction *Daily dose*: 1 dose. The amount of 1 dose was not specified. *Drug to extract ratio*: not known *Standardization*: not known

## Appendix

### Search Terms Used in Different Databases

Cochrane Library, EMBASE, MEDLINE, AMED, CINAHL Plus, PsyINFO ADHD (or attention-deficit/hyperactivity disorder or hyperkinetic disorder or minimal brain dysfunction) and alternative medicine (or complementary medicine or Chinese medicine or kampo or Korean medicine or Oriental medicine or phytotherapy or herbal).

SinoMed (CBM), China Journal Net, WanFang Data—Chinese Databases

1. ADHD and Chinese medicine (in Chinese).
2. Attention-deficit/hyperactivity disorder (in Chinese) and Chinese medicine (in Chinese).
3. Hyperactivity (in Chinese) and Chinese medicine (in Chinese).
4. Minimal brain dysfunction (in Chinese) and Chinese medicine (in Chinese).
5. Hyperkinetic disorder (in Chinese) and Chinese medicine (in Chinese).

Oriental Medicine Advanced Searching Integrated System (OASIS)—Korean Database

1. ADHD.
2. Attention-deficit/hyperactivity disorder (in Korean).

Scholarly and Academic Information Navigator (CiNii), Database of Grants-in-Aid for Scientific Research (KAKEN), Japanese Institutional Repositories Online (JAIRO), Academic Research Database Repository (NII-DBR)—Japanese Databases

1. ADHD and Kampo (in Japanese).
2. Attention-deficit/hyperactivity disorder (in Japanese) and Kampo (in Japanese).
